# Older adults’ perspectives on physical activity during hospitalization: a qualitative interview study

**DOI:** 10.1186/s12877-025-06292-y

**Published:** 2025-09-08

**Authors:** Kristina Dalin Eriksson, Anna-Karin Welmer, Linda Sandberg, Anne-Marie Boström

**Affiliations:** 1 Department of Neurobiology, Care Sciences and Society, Division of Physiotherapy, Karolinska Institutet, Stockholm, Sweden; 2 Aging Research Center, Department of Neurobiology, Care Sciences and Society, Karolinska Institutet, Stockholm, Sweden; 3 Women’s Health and Allied Health Professionals Theme, Karolinska University Hospital, Stockholm, Sweden; 4 Department of Geriatric Medicine, Capio Geriatrik Dalen, Capio Elderly and Mobile Care, Stockholm, Sweden; 5 Department of Neurobiology, Care Sciences and Society, Division of Nursing, Karolinska Institutet, Stockholm, Sweden; 6 Theme Inflammation and Aging, Karolinska University Hospital, Huddinge, Sweden; 7 Research and Development Unit, Stockholms Sjukhem, Stockholm, Sweden

**Keywords:** Older adults, Older patients, Geriatric patients, Hospitalization, Frailty, Sedentary behavior, Physical activity, Exercise, Qualitative

## Abstract

**Background:**

The benefits of physical activity for frail older acutely hospitalized adults are becoming increasingly clear. To enhance opportunities for physical activity on geriatric wards, it is essential to understand the older adult’s perspective.

**Aim:**

The aim of the study was to explore the experiences and perceptions of physical activity among older adults during hospital stays on a geriatric ward.

**Method:**

This was a qualitative interview study with an exploratory interview design, where data were collected through semi-structured individual face-to-face interviews with 20 hospitalized older adults aged 75 years and older. The interviews were transcribed verbatim, and the material was analyzed inductively using qualitative content analysis.

**Results:**

An overarching theme *Barriers and enablers related to the environment*,* personal adaptation*,* and emotional dilemmas influencing changes in physical activity* and three main categories and seven subcategories were identified. The main categories are *Perceiving how context influences physical activity*, *Adapting physical activity to aging and health condition*, and *Balancing emotional dilemmas about engaging in physical activity*.

**Conclusions:**

The results emphasize the need for enhanced communication and personalized care to better support frail older adults in engaging in physical activity during hospitalization. Customized advice and tailored physical activities are key to supporting them in staying active and healthy. Effective strategies, teamwork, and resource allocation are needed to meet these older adults’ needs and develop interventions that ensure proper care and support.

**Trial registration:**

This study did not involve any healthcare interventions on human participants. Data were collected through interviews, and focused solely on exploring experiences and perceptions, with ethical approval obtained for the study.

**Supplementary Information:**

The online version contains supplementary material available at 10.1186/s12877-025-06292-y.

## Background

The global population is aging rapidly, with the number of individuals aged 60 and older projected to grow from 900 million in 2015 to 2 billion by 2050 [1]. In Sweden, the number of people in the population aged over 80 years old is expected to more than double in the coming decades, according to Statistics Sweden [2]. This demographic shift can be seen as a challenge not only for society as a whole but also for individuals, particularly in terms of healthcare, as older people face an increased risk of illness, for example risk of heart failure, neurological diseases, and cancer [3]. As the number and proportion of older people in society rise, so too will the prevalence of frailty and related diseases [4]. Frailty increases the risk of adverse health outcomes in older adults and is associated with declining function, reduced organ reserves, and heightened vulnerability to stressors [4–7]. Since the prevalence of frailty rises with age [5, 8], and the global geriatric population continues to grow, frailty poses an increasing challenge for healthcare systems [4]. Frailty often coexists with sarcopenia, a widespread disorder affecting skeletal muscles that involves a decline in both muscle strength and mass, and is associated to various negative health outcomes [9, 10].

Hospital care entails risks, for example hospital acquired disability, and although hospitalization benefits older adults with regard to acute medical conditions, it often results in low levels of physical activity due to prolonged bed rest or sitting time [11]. Monitoring of mobility capacity during hospitalization revealed that older inpatients spent most of their time (83%) in bed, despite being able to walk independently [4] and a recent study shows that limited mobility is common among older adults in acute care wards, and that these patients perceive a range of complex and overlapping barriers and facilitators to mobilization [12]. This immobilization leads to functional decline, which is the primary hospital-related complication for older adults [13]. Extended immobility quickly speeds up the loss of muscle mass and strength, with older adults potentially losing up to 5% of their muscle strength per day [14]. Studies have shown that more than 30% of older adults experience a loss of independence in activities of daily living following an acute hospital stay [15–17]. In hospitalized older adults, functional decline can occur within days, often due to normal aging combined with bed rest and immobility, which leads to irreversible physiological changes and poor discharge outcomes [5].

To address this issue, and to enhance the capacity of the older adult, health interventions that focus on promoting resources and preventing risks are necessary [8]. Regular exercise is the only intervention consistently proven to enhance physical function, reduce sarcopenia, and improve mood in both frail and non-frail older adults [6]. A recent meta-analysis indicates that in-hospital exercise interventions effectively enhance functional independence and reduce adverse events in older adults [18]. The benefits of physical activity with regard to disease and aging are widely accepted, yet, despite this, exercise is not sufficiently recommended to older adults [19]. Resistance exercise training involves engaging the muscles to work against an external force or weight [20] and is often recommended as a first-line prescription to improve muscle strength and function in older adults with frailty, with guidelines available to support clinicians and practitioners [21, 22].

Duplaga et al. showed in a review that most health interventions were not tailored to older individuals, which can lead to problems with implementation [23]. Older adults may perceive physical activity as a balance between what feels helpful and the risk of injury to an aging body [24]. Older individuals value their mobility and independence in daily activities very highly, even more so than longevity itself [25]; physical activity can also contribute to a more meaningful day – even in the later stages of life [26]. Another study, involving older adults in a day care unit, found that those with multiple long-term conditions, frailty, and recent health decline often lack awareness and understanding of resistance exercise. However, despite facing various barriers, the older adults expressed a willingness to engage in exercise if they received adequate support [21]. Collaboration, therefore, is needed to create and implement effective strategies, including education, to increase the recognition of, and knowledge about, exercise and to encourage this group to participate [21].

To conclude, there is limited knowledge about older adults’ experiences and perceptions on physical activity during hospitalization. To optimize the motivation of older adults to engage in physical activity during hospitalization and compliance, it is important to understand their experiences and preferences to allow interventions to be tailored to meet their individual needs. The aim of this study was to explore the experiences and perceptions of physical activity and inactivity among older adults during their stay on a hospital geriatric ward.

## Methods

### Study design

A qualitative method with an exploratory interview design was used in this study to capture the complexity of older adults’ individual experiences and perceptions, as well as contextual factors. The material was analyzed inductively through content analysis guided by Graneheim and Lundman [27–29]. The COREQ checklist for reporting qualitative research was followed [30] (see Additional File 1).

### Settings

The participants were recruited from a geriatric ward in a university hospital in Sweden. This ward admits a diverse range of acutely hospitalized older adults, primarily with diagnoses related to the respiratory, circulatory, and genitourinary systems [31]. In 2022, when the interviews were completed, the average age of patients on the geriatric ward was 82 years (median 83) and their mean length of stay was 6.3 days, as reported by hospital staff. This study was conducted alongside the research project *Preventing functional decline in acutely hospitalized older patients (PREV FUNC)*, which has been evaluated for feasibility in geriatric wards across three Swedish hospitals in 2022; detailed descriptions of the trial are provided in previous publications [32, 33].

### Sampling and recruitment of participants

All the older adults were enrolled during a hospital care episode on a geriatric ward. The inclusion criteria for the study were hospitalized older adults aged 75 years or older, admitted to one of five geriatric medical wards at a university hospital in Sweden. The exclusion criteria were inability to participate in an interview due to cognitive impairment and serious medical conditions contraindicating exercise, as assessed by a physician. A purposive sampling method was employed to ensure a wide range of perspectives and experiences [34] to capture a rich variety of the participants’ views. Frailty was a common characteristic of the study population given the clinical context, and the sampling strategy was structured to reflect a broad spectrum of frailty levels [35] among participants. A registered nurse at the hospital, not involved in direct patient care to avoid influencing participation, was tasked with identifying suitable informants based on the inclusion and exclusion criteria. The patients were asked if a researcher could approach them about participating in the study. The patients who met the criteria were then invited to participate by the first author who gave verbal and written information about both the study and the ethics review board approval. In total, 21 patients were invited to participate, of whom one declined, and a total of 20 patients were therefore included. Recruitment continued until the first author recognized recurring statements within the collected data material [34]. The interviews were carried out over a period of one year (2021–2022) due to outbreaks of COVID-19 and norovirus on the ward, which resulted in restrictions on in-person meetings.

### Data collection

Data were collected in this qualitative study through 20 face-to-face interviews, guided by a semi-structured interview guide with ten open-ended questions. The interview guide allowed the interviewer to further explore reflections or topics that came up naturally during the conversation with the older adult. The guide was developed by the first author and revised with input from the second and last authors. The guide was subsequently evaluated through two pilot interviews, which helped refine the wording and flow of the questions to enhance clarity and ensure they were easily understood by the participants. The open-ended questions concerned *what* and *how*; for example: “How do you perceive that the hospital environment and the ward’s routines have affected your ability to move and be physically active?”. The interview guide developed for this study is provided as Additional File 2. The interviews were audio-recorded for analysis. The first author conducted the data collection, which included interviews, audio recording, and transcribing. Confidentiality was ensured by using a code number for each interview transcript. The interviews had a median duration of 13 min (range 7–21). The intention was to conduct interviews in a private setting, with participants being offered a separate room for the interview. The patient rooms varied from single to shared rooms with 2–4 beds and most older adults preferred to stay in their own rooms for the interview, except for one who chose the corridor. Information regarding frailty, based on the Clinical Frailty Scale (CFS), was extracted from the patient records by a registered nurse and reflects the patient’s condition prior to the hospital stay. CFS is a scale from 1 to 9, where 1 represents very fit and 9 terminally ill [35]. Other demographic data, including age, gender, social situation, medical condition, and length of stay, were also retrieved from the patient record by the registered nurse.

### Data analysis

The analysis process was guided by Graneheim and Lundman’s framework for qualitative content analysis [27–29] and led by the first author. Microsoft Word was used to manage the data. The study primarily focuses on the manifest content of the material, but also examines how the categories are connected under a broader concept, which results in an overarching theme [28]. Initially, the recorded interviews were transcribed verbatim, and the first phase in the analysis included reading the transcripts thoroughly several times to obtain an overall sense of the texts and their content. Meaning units were identified and labeled with codes describing the sentence content, intending to address the purpose and context. In the second phase, the codes were compared to find similarities and differences and were then sorted into subcategories with closely related codes, primarily displaying the text’s manifest content. Subsequently, main categories were developed by organizing and bringing together related subcategories. The analysis process was iterative, with codes, subcategories, and categories being continuously refined throughout the process. In the final phase of the analysis, the categories were connected under a broader overarching theme, abstracted and synthesized to reflect a deeper understanding. A third of the interviews were read in full text by the third author and analyzed separately, and the results were compared and discussed until consensus was reached. There was continuous dialogue between all authors throughout the analysis process to arrive at a shared understanding of the content of the analysis.

### Reflexivity

The research team consisted of two registered nurses, KDE (MSc) and AMB (PhD), a physiotherapist, AKW (PhD), and an occupational therapist LS (PhD), all female with experience in qualitative studies. This interdisciplinary team provided a broad knowledge base. Clinical care, physical activity, and functional independence were key areas of expertise that enabled a multifaceted approach to the data, supporting rich discussions and a comprehensive understanding of the participants’ experiences. The team structure offered a strong foundation, with researchers exchanging their perspectives and interpretations throughout the iterative analytical process. None of the authors were involved in the participants’ care or rehabilitation.

## Results

### Description of participants

Of the 20 participants, 12 were women and 8 were men. The median CFS score was 5 (ranging from 3 to 7). The median age was 86.5 years (ranging from 75 to 94), the length of hospitalization varied from 2 to 38 days (median 8), 75% of the participants were discharged to their own homes, 40% lived with spouse, and 60% lived alone. The reason for admission refers to the primary medical condition leading to hospitalization, categorized as respiratory and circulatory disorders (20%), musculoskeletal disorders and injuries (20%), genitourinary and infectious disorders (20%), and general symptoms/deconditioning (40%) (see Table 1).

**Table 1:** Characteristics of the study population

Description of participants (*n* = 20)	
Age in years, median (range)	86.5 (75–94)
Gender, female, n (%), male, n (%)	12 (60), 8 (40)
Length of stay in days, median (range)	8 (2–38)
CFS^1^, median (range)	5 (3–7)
Medical condition, reason for admission	
Respiratory and circulatory disorders, n (%)	4 (20)
Musculoskeletal disorders and injuries, n (%)	4 (20)
Genitourinary and infectious disorders, n (%)	4 (20)
General symptoms/deconditioning, n (%)	8 (40)
Discharged to own home, n (%)	15 (75)
Living alone, n (%)	12 (60)
Living with spouse, n (%)	8 (40)

^1^*CFS *Clinical Frailty Scale by Rockwood and Theou (2020)

**Fig. 1:**
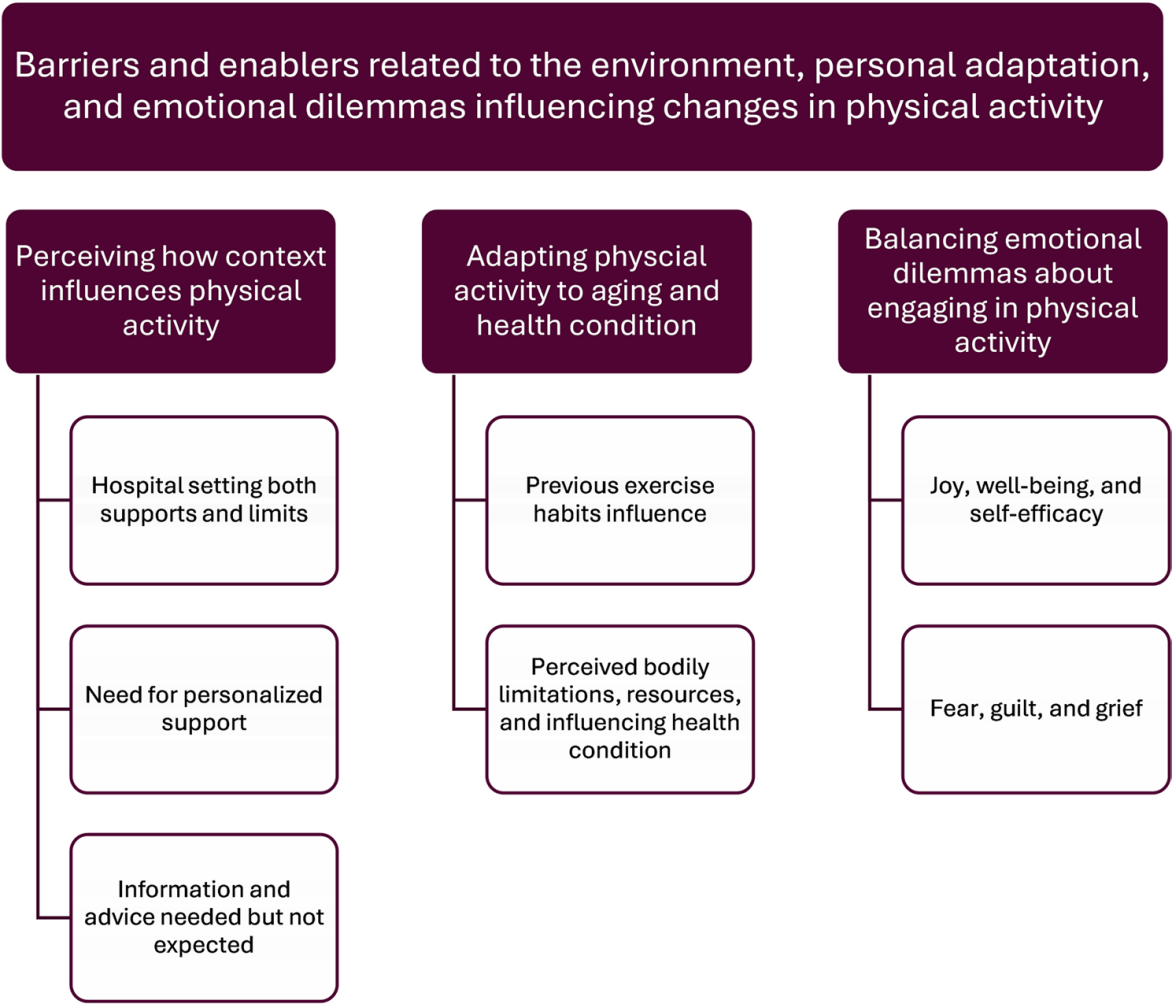
Theme, categories, and sub-categories

The results derived from the analysis consist of an overarching theme, three main categories, and seven sub-categories (see Fig. 1).

### Barriers and enablers related to the environment, personal adaptation, and emotional dilemmas influencing changes in physical activity (theme)

The overarching theme *Barriers and enablers related to the environment*,* personal adaptation*,* and emotional dilemmas influencing changes in physical activity* relates to frail older adults describing changes in physical activity influenced by personal and environmental factors. The older adults referred to physical activity by mentioning a range of activities, including exercises they had performed earlier in life, daily activities including gardening, walking, hiking, and other forms of everyday physical activity, but also exercises and activities undertaken during their hospitalization. They reflected on how to make physical activity possible in relation to their aging bodies, the obstacles they encounter, and the conflicting emotions that arise.

### Perceiving how context influences physical activity (category)

This category includes three subcategories that explore contextual barriers and enablers influencing physical activity among older adults during hospitalization: *Hospital setting both supports and limits*,* Need for personalized support*, and *Information and advice needed but not expected.*

### Hospital setting both supports and limits (sub-category)

The hospital environment both supports and limits physical activity. Walking in the corridors was the primary opportunity for physical activity identified by the older adults. Environmental barriers, including restrictions due to COVID-19 or norovirus outbreaks, often resulted in immobility. The older adults valued physical activity for its health benefits and as a means of managing their health conditions. They mentioned the long, wide corridors with handrails along the walls as an environmental support and a means of engaging in physical activity through walking, but their ability to engage in physical activity was sometimes also constrained by the environment. They wanted to improve their physical abilities and expressed a desire to walk further within the hospital but faced obstacles — including limited staff availability to provide support and restrictions on corridor use due to infection control measures. In addition, ongoing medical examinations and procedures often reduced the opportunities for walking in the corridor.

*“…I’ve got some exercise stuff I do on my own*,* so I go out into the corridor*,* and there are handrails there*,* and then I kick my legs a bit and stuff like that*,* you know.” (Female*,* 94)*.

*“Well… the thing is… there’s pretty low activity when you’re in a hospital setting*,* you sit and lie down and… you need to move more… Yeah*,* the [hospital environment] really limits your possibilities to move in many ways…” (Female*,* 87)*.

*“There’s not much I can do except walk back and forth in the corridor. I haven’t asked if I can go outside*,* no*,* but I don’t plan on doing that either.” (Female*,* 86)*.

### Need for personalized support (sub-category)

The older adults described that receiving personalized support and technical aids to facilitate physical activity is of particular importance. The older adults mentioned various forms of support that were necessary to facilitate their ability to engage in physical activity and movement. They described needing personal support to be physically active in the hospital environment, i.e., assistance from staff or caregivers to feel safe while walking, especially if they felt unsteady.

*“I need someone beside me because I feel unsteady*,* I lose my balance*,* and I don’t want to fall and hit my head again.” (Female*,* 85)*.

*“I am completely exhausted – I can hardly even get to the bathroom from here without support.” (Male*,* 92)*.

On the other hand, they described that the limited availability of staff prevented them from receiving the support they needed. The older adults expressed this as a perceptible barrier, compounded by the older adults’ reluctance to seek assistance, perceiving that caregivers were already overwhelmed with other responsibilities.

*“… how could they [healthcare staff] manage that with a thousand and one other things to do? I’m not asking for that.… but this time I’ve spent a lot of time in bed. Too much.” (Female*,* 92)*.

The older adults expressed a need for individually tailored and customized support for physical activity during hospitalization, emphasizing the importance of staff adopting a more creative and flexible approach based on each older adult’s specific needs and situation.

*“…I’d like a bit more of a creative approach to the whole thing … like thinking outside the box*,* you know? But I guess you can’t really ask for that*,* can you?” (Male*,* 84)*.

In addition, the older adults mentioned that access to technical aids, like walkers and canes, was needed to enable movement.

### Information and advice needed but not expected (sub-category)

The older adults expressed differing views on information and advice from healthcare professionals regarding physical activity and exercise, perceptions that were often shaped by their previous experiences. The older adults experienced that they did not receive advice related to physical activity in the context of illness or aging. They expressed uncertainty about what types and amounts of activity were safe and beneficial during their hospital stay, leading to hesitation or avoidance of movement, so therefore needed more information and advice. Despite this, they did not expect or actively request such advice.

*“…honestly*,* I haven’t actually thought about it*,* but no one has said a word about anything like that.” (Male*,* 78)*.

However, they appreciated the encouragement they received from staff when they were active — whether getting out of bed or walking in the corridors. The older adults recalled receiving specific advice from physiotherapists, although in general, information was sparse.

*“…they often say something encouraging when they see you in the corridors*,* like ‘it’s good that you’re up and about’… and they point out my posture*,* because it’s terrible. I should work on that more.” (Female B*,* 78)*.

### Adapting physical activity to aging and health condition (category)

The older adults described that the inevitable, gradual changes in the individuals’ physical conditions in relation to activity posed challenges in life. This category explores how participants adapt their physical activities to accommodate their reduced capabilities and includes two subcategories: *Previous exercise habits influence* and *Perceived bodily limitations*,* resources*,* and influencing health condition*.

### Previous exercise habits influence (sub-category)

The older adults described their past engagement in physical activity as being a large part of their daily lives, and included walking, household chores, gardening, and even movement within their former occupation. Running, walking the dog, swimming, cycling, tennis, group exercise sessions, and strength training were also mentioned as forms of physical activity.

*“I’ve been going swimming and cycling*,* all my life. And I’ve felt good because of it. Every day. I wish everyone did that.” (Female*,* 91)*.

When the older adults discussed their thoughts about exercise and training, they often related this to experiences from earlier years, and they described how they had been active earlier in life but that their physical activity had diminished with age.

*“…then I’ve played both tennis and done archery and all sorts of things like that … I have been very active my whole life.” (Male*,* 85)*.

As older adults, their activity mainly consisted of walking, which they continued doing for as long as their health allowed. Environmental factors during winter, for example the risk of slipping on ice, further restricted their ability to engage in outdoor activities during that season. They reminisced about their more active past and felt frustrated by the decline in their physical abilities.

### Perceived bodily limitations, resources, and influencing health condition (sub-category)

The older adults focused on the various physical limitations that impede their ability to remain active during their hospital stay. These challenges became more pronounced as their health deteriorated, forcing them to confront the inevitable decline in their physical function. Pain, fatigue, reduced stamina, balance issues, reduced mobility, muscle weakness and stiffness were barriers mentioned, leading to a gradual decrease in physical activity.

*“…I’ve been sitting a lot due to knee pain … I’ve received all the help I can get here*,* but I don’t walk much because of it. I sit a lot and… it’s mostly sedentary…” (Female*,* 87)*.

The older adults described being aware of their body, and its capabilities and weaknesses. They described mild to severe movement limitations, varying among the individuals, but they felt that several factors hindered them during their hospital stay.

*“… I derive no pleasure from it [physical activity] at all because I have balance difficulties*,* dizziness*,* and I’m tired*,* so… from being a necessity and a source of strength to it becoming tiresome and exhausting and just a duty…” (Male*,* 92)*.

The older adults experienced that they adapted their physical activity over time in response to aging and declining health. They noticed changes in their body, both physically and functionally, in relation to their health condition and aging.

*“I have a walker and so on*,* but I haven’t had the energy because my muscle mass has completely disappeared*,* so I can barely walk a step.” (Female*,* 91)*.

They described a gradual reduction in activity, sometimes interrupted by acute health events that necessitated hospitalization. Despite feelings of sadness over their declining physical abilities, they made efforts to continue moving, recognizing the importance of staying active.

*“…previously*,* I used to walk in the hallway and… I also did these squats as I mentioned*,* but this time it’s not possible… it’s terrible… yes*,* they… it’s not going upwards*,* at my age*,* it only goes downwards*,* the question is just how fast.” (Male*,* 92)*.

They also acknowledged the benefits of physical activity for health and well-being and described how it strengthened their own resources, both physically and mentally.

*“But*,* you do feel better [when exercising]*,* and I notice… well*,* I understand now after retiring*,* I also felt really good then*,* because I was pretty active and went to work out regularly.” (Female A*,* 78)*.

*“…that’s what you should do because it’s half the remedy*,* using all the muscles and everything*,* if there are any left*,* but you have to take care of what you have.” (Male*,* 87)*.

The older adults accepted aging as an unavoidable process and focused on managing it to the best of their ability, even as they faced the reality of their diminishing physical capacity. They described this acceptance as taking a practical approach, shaped by their life experience and understanding of their own situation. The older adults described how they adapted their routines and expectations, from performance to maintaining independence and well-being in everyday life. The older adults expressed frustration over lost abilities but emphasized the importance of staying active within their current limitations — whether through shorter walks, gentle exercises, or simply getting out of bed. This process of adjustment was accompanied by a desire to preserve dignity and a sense of purpose, even as physical strength declined.

*“…in some way I’ve gained a kind of insight into*,* well*,* the finiteness of life. I won’t live forever and… the problems I have are also related to the fact that life is coming to an end*,* illness is a sign of that.” (Female B*,* 78)*.

The older adults also described that health-related factors limited their ability to be active during their time in hospital. Their health condition varied in severity, for example cardiovascular diseases, infections, cancer, fractures, reduced general health status, and injuries sustained from falls. They were also limited by a range of other ailments, including pain, fatigue, lack of energy, and dizziness. As a result, they felt that it was not easy to engage in physical activity during their hospital stay.

*“I’ve probably moved as much as I could until I had this troublesome femur fracture*,* which put an end to most of that.” (Male*,* 92)*.

*“I do try… even though I’m in pain*,* but you know… of course it’s hard*,* it’s easier to just stay in bed*,* I have to admit that. Even though I — I want to get up*,* I really do*,* but I want to get up feeling happy and energetic*,* not ugh*,* not like that. But that’s how it is almost every day*,* yeah*,* so it’s tough.” (Female A*,* 78)*.

### Balancing emotional dilemmas about engaging in physical activity (category)

The older adults expressed complex and varied emotional experiences associated with physical activity in general and this category includes the following two subcategories: *Joy*,* well-being*,* and self-efficacy*, and *Fear*,* guilt*,* and grief*.

### Joy, well-being, and self-efficacy (sub-category)

The older adults emphasized the positive emotions and importance of exercise for both mental and physical health, underlining its value for overall wellness, self-efficacy and life in general. They noted that exercise brings a sense of well-being and a longing for the ability to move more freely.

*“I see it [physical activity] as something really positive*,* because I know it’s good for me. It almost makes me feel young again.” (Female*,* 85)*.

Physical activity was also valued for its social benefits, as it provided opportunities for interaction with others, whether through group activities such as gymnastics, dance, senior fitness class, or simply being able to have a conversation when walking, which was a source of joy. The social aspect of exercise was considered to be valuable, making physical activity more meaningful and enjoyable.

### Fear, guilt, and grief (sub-category)

The older adults mentioned experiencing increased negative emotions toward physical activity as they aged. While they recognized the benefits of staying active, the older adults also found exercise challenging, burdensome, and even frightening due to the perceived risk of devastating injuries, particularly from falling. As a result, physical activity was not always associated with beneficial effects. Instead, it was also linked to fear and insecurity, which overshadowed the potential health gains, which contributed to avoidance behaviors and reduced motivation in their later years.

*“I think it [physical activity] is awful. I don’t like it*,* but you have to do it for your health” (Female*,* 85)*.

The older adults experienced feelings of guilt and self-blame, and they described themselves as lazy or unmotivated because of their lack of physical activity.

*“It’s laziness that stops me*,* … it’s really hard at this age to keep active*,* really hard.” (Male*,* 79)*.

*“…but I’m trying really hard to stay active … [anything else] would make me feel ashamed.” (Male*,* 77)*.

They shared feelings of grief over their diminished ability to engage in physical activity as they once had, with activities that were once enjoyable now feeling like tiresome, burdensome tasks. They described physical activity as something they forced themselves to do, unable to derive any pleasure from it anymore and expressed difficulty finding the motivation to move. This applied both to when they were in the hospital bed and at home on the couch.

## Discussion

The aim of the study was to explore the experiences and perceptions of physical activity among older adults during hospital stays on a geriatric ward. The study resulted in the overarching theme: *Barriers and enablers related to the environment*,* personal adaptation*,* and emotional dilemmas influencing changes in physical activity* with three main categories *Perceiving how context influences physical activity*, *Adapting physical activity to aging and health condition*, and *Balancing emotional dilemmas about engaging in physical activity*. For frail older adults, acute hospital care is often essential but poses risks due to reduced physical activity leading to functional decline and negative health outcomes [4, 5, 36], while regular exercise is shown to improve physical function regardless of frailty [6]. This study shows that older adults perceive that contextual aspects impact their ability to engage in physical activity. It highlights that older adults experience the hospital environment, the availability of personalized support, and ability to adapt to be important factors influencing their opportunities for physical activity. The perceived information gap, alongside feelings of insecurity, underscored the importance of proactive, personalized communication from healthcare providers to support and motivate older patients in remaining physical active during hospitalization. Sourdet et al. identified concerns in patients with preventable, treatment-induced disabilities, including low mobilization, highlighting the need to educate healthcare professionals about older adults’ specific needs and the risks of hospitalization-associated disability as a potential outcome of care [37].

Additionally, older adults may perceive physical activity as being more strenuous than previously, due to reduced bodily function. A recently published article suggests that current measurement methods of physical activity using the *Metabolic Equivalent of Task* concept in older adults may underestimate the intensity of their activities, and the way we currently classify physical activity levels should be updated for older adults using, for instance, oxygen uptake (VO2 Reserve) [38]. This seems to be consistent with how the older adults in this study experience the gradual deterioration of their bodies and their difficulty in maintaining their previous levels of physical activity. Aging was perceived as an inevitable process affecting their ability to be physically active. They experienced that immobility during hospitalization stemmed mainly from resource limitations. They described a balancing act between difficulties and meaningfulness, where barriers were considerable and physical activities in daily life needed to be adapted to their abilities. However, they also described the advice given about physical activity as not being particularly helpful. When designing physical activity programs for older hospitalized individuals, it is necessary to consider not only their information needs but also their perceived barriers, support requirements, motivational factors, available resources, and the importance of social interaction. It has been shown that physical activity during hospital stays reduces functional decline in older adults. A meta-analysis found that as little as 25 min of slow walking daily improves functional capacity and reduces adverse events, with optimal results at around 50 min of training per day, and that bed rest is less safe than staying active during acute hospital stays [13].

The older adults also expressed an emotional dichotomy related to physical activity. While it brought joy over benefits, well-being and a sense of vitality, it also triggered fear of injury, guilt over inactivity, and grief over lost abilities, which lead to hesitation and anxiety. This emotional dilemma illustrates how physical activity in later life is not merely a health-related behavior but a personal experience, shaped by changing bodies and the balance between ability and limitation. The older adults in this study described aging as an inevitable process that they needed to accept and come to terms with. They expressed sadness over the decline in their physical function but attempted to adapt through some form of movement, as they understood the importance of physical activity. This aligns well with Åhlund et al. who noted that patients had gradually adapted to their new situation and accepted that their capacity for physical activity and exercise had changed due to their aging bodies [24]. However, older adults are a heterogeneous group, and Hägg et al. emphasize that regardless of chronological age and comorbidities, some age faster and become more vulnerable and susceptible to illnesses than others [39]. Maintaining skeletal muscle mass and function during aging is necessary for preserving quality of life and health [40]; older adults need, therefore, to be assessed based on the concept of frailty, and physical activities should be tailored to their individual circumstances and needs. Frailty assessment helps clinicians tailor care to each older adult’s health status and both frailty and sarcopenia are conditions that can be mitigated through regular physical exercise [7]. This study contributes to a further understanding of older adults’ experiences and perceptions of physical activity during hospitalization on a geriatric ward. Overall, the findings describe how health conditions and aging clearly impact the older adult’s experience and perception of physical activity.

It is well known that healthcare is undergoing a rapid change, with older adults having increasingly complex needs, hospital stays becoming shorter and demands for efficiency increasing [4, 6, 41]. Older adults often require a higher level of care and attention due to multiple chronic illnesses and reduced mobility, and there is convincing evidence that this patient group needs mobilization and physical activity during hospitalization [42]. In this study, the older adults expressed an interest in engaging in physical activity but reported not receiving the necessary support. Studies show that while nurses have the knowledge, and understand the need for mobilization in older adults [43], basic care such as mobilization is often overlooked in hospital settings due to competing priorities, particularly medical interventions [44]. Making hospitals more age-friendly will require teamwork across different professions and a shift in how we value the care of older adults in hospital [45], but the concept of age/senior friendly hospital care [46] has not yet been implemented in Sweden. Today, it is not clear how older adults will receive support and have their needs for mobilization and exercise met in this environment, and the effectiveness of exercise interventions and healthcare professionals’ perceptions should be further developed. To address these challenges, improved age-friendly strategies and effective teamwork are required to efficiently allocate time and attention across all healthcare professionals, ensuring that each older adult receives the appropriate care and personalized support.

### Methodological considerations

In order to be trustworthy, a qualitative study requires a thorough design. This study has been evaluated using Lincoln and Guba’s criteria for trustworthiness [47]. Securing data from 20 participants was considered to provide ample depth when responses were repeated without new insights [34]. The study was conducted in only one department of one hospital, which is a limitation. All authors participated in the data analysis, with diverse expertise enhancing the trustworthiness of the findings. Peer debriefing strengthened credibility by ensuring that findings were reviewed from multiple perspectives. Conducting interviews in patient rooms may have affected dependability due to the potential for interviews to be overheard but given that the locations were selected by the interviewee, they were deemed acceptable. Some interviews were brief (median duration 13 min), flexibility was required, and the interviews were adapted to accommodate the needs of frail older participants. The interviews were conducted during 2021–2022 when outbreaks of COVID-19 and norovirus occurred, which may have influenced the older adults’ perceptions during their hospital stay. Another limitation was the lack of participant validation, since discharged patients could not review the transcripts. The researcher’s role in this study has been carefully considered and the first author, a registered nurse, reflected on biases to minimize impact. By being aware of, and open about, these aspects, the study aims to enhance the credibility and transparency of the findings. Overall, strengths such as achieving data to the point of redundancy, peer debriefing, and team diversity enhance credibility, but the interview setting and short duration of interviews are limitations to consider.

## Conclusions

The findings indicate that health conditions and aging shape frail older adults’ experiences and perceptions of physical activity, impacting their ability to engage in physical activity during hospitalization. The older adults noted that their views on physical activity evolved over time, influenced by personal health and available support, with emotional factors also playing a role. Frail older adults with comorbidities require tailored advice and support for physical activity during hospital stays, and the hospital environment was perceived as both hindering and supportive. The older adults perceived aging and physical decline as inevitable and expressed uncertainty about the benefits of activity in the hospital, which highlights that clear, personalized communication is essential. Future research should evaluate the effectiveness of exercise interventions and healthcare professionals’ perceptions. Overall, the study emphasizes the requirements for customized interventions to meet older adults’ specific needs, providing valuable perspectives for healthcare providers.

## Supplementary Information

Supplementary Material 1.

Supplementary Material 2.

## Data Availability

The interviews analyzed in this study are not publicly available due to the presence of identifiable patient information. Anonymized data may be made available from the corresponding author upon reasonable request.

## Abbreviations

CFS: Clinical Frailty Scale
COREQ: Consolidated Criteria for Reporting Qualitative Research
