# Supplementary material for: Older adults’ perspectives on physical activity during hospitalization: a qualitative interview study
Source: BMC Geriatr. 2025 Sep 8;25:688. doi: 10.1186/s12877-025-06292-y (PMC12418710; doi:10.1186/s12877-025-06292-y)
Supplement: Supplementary file 2 — Supplementary Material 2. [file 12877_2025_6292_MOESM2_ESM.pdf]

Additional File 2

Interview guide

Questions

1 What comes to mind when I say *physical activity*?

2 What does "being physically active" mean to you?

3 What are your past experiences of being physically active? Describe a physical activity you engaged in 5-10 years ago. How often were you physically active then? What are your thoughts about this?

4 What opportunities for physical activity have you had at home during the last month before hospitalization? Describe a physical activity you engaged in during that time. How often were you physically active? What are your thoughts about this?

5 How do you feel your current health condition has affected your ability to move and be physically active? What are your thoughts about this?

6 How do you feel the hospital environment and the ward's routines have affected your ability to move and be physically active? What are your thoughts about this?

7 What do you think is expected of you in terms of rest or movement/physical activity here in the hospital? What are your thoughts about this?

8 What could motivate you to get up and move around here in the hospital?

9 Healthcare staff experience that patients in hospital spend a lot of time in bed or sitting in a chair. Is this something you recognize yourself? What do you think about this? What do you think might be the reasons for this?

10 Can you describe any advice about physical activity and movement that you have received from healthcare professionals? What do you think about this? What information would you like to have received?
